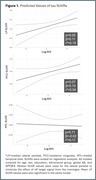# Associations between sleep apnea and tau in cognitively unimpaired older adults

**DOI:** 10.1002/alz.095324

**Published:** 2025-01-09

**Authors:** Victoria R Tennant, Amaryllis A Tsiknia, Carrie B. Peltz, Noelle Lee, Maxwell W Hand, Yue Leng, Katie L Stone, Susan Redline, Sid E. O'Bryant, Meredith N Braskie, Kristine Yaffe

**Affiliations:** ^1^ University of Southern California, Los Angeles, CA USA; ^2^ Stevens Neuroimaging and Informatics Institute, Marina del Rey, CA USA; ^3^ Stevens Neuroimaging and Informatics Institute, Marina Del Rey, CA USA; ^4^ NCIRE‐The Veterans Health Research Institute, San Francisco, CA USA; ^5^ Stevens Neuroimaging and Informatics Institute, Los Angeles, CA USA; ^6^ University of California, San Francisco, San Francisco, CA USA; ^7^ California Pacific Medical Center Research Institute, San Francisco, CA USA; ^8^ Harvard T.H. Chan School of Public Health, Boston, MA USA; ^9^ Brigham and Women’s Hospital, Harvard Medical School, Boston, MA USA; ^10^ University of North Texas Health Science Center, Fort Worth, TX USA; ^11^ Departments of Psychiatry and Behavioral Sciences, Neurology, and Epidemiology, University of California San Francisco, San Francisco, CA USA

## Abstract

**Background:**

Older age is associated with sleep disruptions and the aggregation of pathological tau in the medial temporal lobe (MTL). Associations between sleep disruptions and the progression of Alzheimer’s disease (AD) have been widely demonstrated. However, data addressing the association of objectively measured sleep apnea to tau deposition beyond the MTL in cognitively unimpaired older adults are limited.

**Method:**

We included 357 cognitively unimpaired participants from the DORMIR sleep study, an ancillary study of HABS‐HD, with available information on the apnea‐hypopnea index (AHI; number of respiratory events with ≥3% desaturation divided by the estimated all‐night sleep time) derived from a home sleep apnea test (WatchPat, Itamar, Medical), Aβ (florbetaben), and tau (PI‐2620) PET scans. We calculated Aβ and tau standardized uptake value ratio (SUVR) using FreeSurfer (v5.3.0) derived regions, with whole cerebellum and posterior gray matter of the cerebellum as the reference regions, respectively. Between‐group differences for high AHI (AHI≥15) and low AHI (AHI<15) were assessed using t‐tests for continuous variables and χ2 tests for categorical variables. We evaluated the independent effect of log‐transformed AHI on continuous global Aβ and tau in separate linear regression models, adjusting for age, sex, education, ethnoracial group, and *APOE4* carrier status. When predicting tau, we also included continuous global Aβ as a covariate.

**Result:**

Median lateral parietal tau SUVR was significantly higher in the high AHI group (p = 0.04). Higher AHI was associated with higher median lateral parietal tau SUVR (p = 0.02, std.β = 0.11) and higher posterior cingulate tau SUVR (trend; p = 0.07, std.β = 0.10), but not with MTL tau SUVR (p = 0.71, std.β = ‐0.02) (Figure 1). AHI was not associated with global Aβ SUVR (p = 0.24, std.β = 0.06).

**Conclusion:**

Past work has linked sleep apnea to impaired Aβ clearance. Intriguingly, our findings link higher AHI to more tau in the lateral parietal and posterior cingulate cortex, independent of Aβ burden, suggesting a separate pathway by which sleep apnea may exert effects on the aging brain. If replicated in longitudinal studies, this may have important implications for the development of sleep health guidelines and AD prevention strategies.